# Examining the Urban and Rural Healthcare Progress in Big Cities of China: Analysis of Monitoring Data in Dalian from 2008 to 2017

**DOI:** 10.3390/ijerph17041148

**Published:** 2020-02-12

**Authors:** Yukun Qiu, Wei Lu, Jianke Guo, Caizhi Sun, Xinyu Liu

**Affiliations:** 1 School of Architecture and Arts of Dalian University of Technology, Dalian 116081, China; qiuyukun@mail.dlut.edu.cn (Y.Q.); liuxinyu1213@mail.dlut.edu.cn (X.L.); 2 Research Center of Ocean Economy and Sustainable Development of Liaoning Normal University, Dalian 116029, China; guojk2012@lnnu.edu.cn (J.G.); suncaizhi@lnnu.edu.cn (C.S.)

**Keywords:** urban and rural disparity, healthcare system, GIS, weighted TOPSIS model, obstacle model

## Abstract

How to effectively reduce the disparity between urban and rural medical healthcare has become a major global concern. In China, the government has issued a series of reform measures to address the gap between urban and rural medical care. To explore the impact of China’s medical system reforms in improving health services in urban and rural areas and understand the factors promoting and hindering progress, we evaluated the healthcare system in Dalian City, China, from 2008 to 2017. The weighted TOPSIS (technique for order preference by similarity to ideal solution) model was used to assess the development of the healthcare system in the different districts and employed the obstacle model to identify and analyze indicators that hinder progress in health services. Using the local spatial clustering function, we categorized the districts in terms of the hindrance type that significantly hamper the growth of the healthcare system. Our results show the healthcare system in Dalian’s urban areas has steadily increased, while development in rural areas has been erratic. Although the urban–rural healthcare disparity has narrowed distinctly, sustained progress is not guaranteed. Based on the location theory, residents in urban areas are more affected by economic factors, while those in rural areas are more influenced by time considerations. When initiating healthcare reforms in urban areas, the impact of varying land prices and per capita disposable income should be considered. For rural areas, constructing more medical institutions to reduce the impact of time costs should be considered. We also found different factors that hinder the growth of the healthcare system for urban and rural areas. To address these impediments to progress, urban areas should pay more attention to coordinated development, while rural areas should address specific concerns based on local needs and conditions. More research on the progress in medical reform is crucial to provide reference and policy-guidance for countries facing similar concerns.

## 1. Introduction

A new medical reform policy was officially started in 2009 by the Chinese government aimed at upgrading the existing medical system and improving the national health environment. In 2010, the World Bank released the report, *China Medical Reform Policy Recommendations*, with regard to China’s new medical reform policy [1]. The report specified that China should narrow the gap between urban and rural areas as one of its main targets for the next five to ten years. In 2018, the World Bank, the World Health Organization, the Ministry of Finance, the National Health and Family Planning Commission, and the Ministry of Human Resources and Social Security jointly issued the report, *Deepening China’s Medical and Health System Reform and Building a Value-Based Quality Service Delivery System*, detailing recommendations for China’s Medical Reform [2]. It reiterated the importance of narrowing the urban–rural gap and the need to coordinate urban and rural medical development.

According to the World Bank and the World Health Organization, about half of the world’s population lacks essential medical services [3]. Compared to many western countries, China’s urban–rural relations have some unique characteristics in terms of formation mechanism, evolution process, and countermeasures. As the most populous country in the world, China has introduced a series of policies to promote the coordinated development of urban and rural health care systems since the implementation of the new medical reform policy. This included making medical insurance more accessible [4,5], increasing the proportion of medical reimbursements [5,6], and reducing medical costs [6,7]. By comparing the medical data of 2017 and 2018, we found that the total number of medical treatments in China’s medical and health institutions reached 8.31 billion in 2018, an increase of 130 million (1.6%) over the previous year. The number of patients admitted to hospitals was 254.53 million, an increase of 10.17 million (up 4.2%) from the previous year, and the annual hospitalization rate was 18.2%. The number of visits to rural health centers was 1.12 billion, which increased by 100 million from the previous year. The number of hospital admissions decreased by 630,000 from the previous year, which stood at 39.84 million. Doctors were responsible for 9.3 consultations per day, and the average length of stay (ALOS) in hospitals was 1.6 days, with a bed occupancy rate of 59.6% and an average hospitalization day of 6.4 days. For rural health centers, the physicians’ workload decreased slightly in 2018, with the bed utilization rate decreasing by 1.7%, and the ALOS in hospitals was extended by 0.1 days. The per capita hospitalization expenses decreased by 2.4 percentage points (42.2%), and the medical reimbursement rate in the rural population was 70%. Overall, China’s health insurance covers more than 1.3 billion urban and rural residents. Therefore, aside from closing the gap between its urban and rural healthcare systems, China is pursuing reforms to lower medical costs and improve the quality of its healthcare services and infrastructure. In addition to reducing the medical disparity between urban and rural areas through direct medical reform initiatives, the Chinese government has promoted economic development in rural areas, increased the proportion of government expenditure for medical construction in rural areas, and created a more conducive growth environment for the pharmaceutical industry.

The World Bank and the World Health Organization regularly publish global health surveillance reports to measure the proportion of people with access to quality essential health services. The reports collect data from different sources, including the World Bank [3], the World Health Organization [8], and the United Nations [9]. These reports have been used in different countries, such as South Africa [10], Iran [11], Thailand [12], and South Korea [13], for a variety of research topics, including the examination of the impact of urban and rural medical gaps. And while there have been several studies exploring the disparity in its urban–rural healthcare systems, there still is a significant knowledge gap on the subject matter. An extensive bibliographical analysis of the progress in the healthcare industry has shown that most of the research had been focused on analyzing institutions [14,15,16] and macro data [17,18,19]. In particular, we found a lack of detailed research on the urban–rural medical gap on a city-level using a multi-attribute evaluation system. Previous studies used only single indicators, such as population, economics, and medical resources, in analyzing the healthcare systems. For example, Alghnam et al. [17] used a population-based analysis of how traumatic injuries affect medical expenditures in the United States. Helt, E.H. [20] and Fairchild, A.L. [21] used economic models and theories to analyze the impact of economic development on the medical security system. Using communities as a research unit, Hibbard, J.H. et al. [22] compared the differences between residents’ self-care and medical care. Linde, A. [23] analyzed the impact of improvements in medical resources on criminal behavior in Germany from 1977 to 2001. While these studies were able to explain some effects of selected variables on the healthcare systems, they provided only a limited understanding of the complex dynamics of the industry since they failed to combine multiple factors in the analysis.

The main objective of this study is to investigate the emerging trends in urban and rural healthcare development and identify significant factors that may hinder improvements in urban and rural health services. To overcome intrinsic and methodological limitations in current studies on healthcare assessment, we developed an approach integrating the analytic hierarchy process (AHP) and entropy weight method (EWM) into the technique for order of preference by similarity to ideal solution (TOPSIS) model. A comprehensive weight model is developed using the combined weights from AHP (providing subjective weight) and EWM (providing objective weight) of each indicator in the regional medical evaluation system. Together with the TOPSIS model, we used the obstacle degree model to calculate the contributions of each index through the degree of hindrance to health care. Our study area is the Chinese city of Dalian, a major port city on the Liaodong Peninsula, situated at the southern tip of Liaoning Province. Much of the datasets used in the study were derived from the Dalian Statistical Yearbook and the Dalian Health Bureau monitoring site, which provide the most comprehensive, official data for the city’s healthcare system. It includes demographic, economic, and social indicators, which we used to establish the regional medical evaluation system. The approach developed in this study can be used as a reference in assessing healthcare improvements in cities, particularly in evaluating disparities between urban and rural progress.

## 2. Methods

### 2.1. Study Design and Data Collection

Dalian is one of the earliest cities to implement reforms in its urban and rural medical system in China. Research on its current healthcare status would provide a reference for other cities facing similar problems. In selecting the healthcare indicators that would be used to establish the evaluation system, we based our approach on previous studies that also dealt with establishing assessment indicators. Our approach utilizes an indicator system based on demographic, socio-economic, and medical data. Previous TOPSIS models have overlooked how the different indicators may have varying effects on the research object [24,25]. To make the research results more realistic, we employed the comprehensive weight model, which was used to obtain the overall weights of each index. We used the weighted TOPSIS model to calculate the scores of medical undertakings in the various regions of Dalian from 2008 to 2017. Based on the contributions of each indicator, we identified the significant indicators that promote the development of medical care in various regions. Based on the results of the obstacle model calculation, we determined the indicators that hinder progress in healthcare services in various regions of Dalian. And using the local spatial clustering function in ArcGIS10.5 (Environmental Systems Research Institute, Redlands, CA, USA), each region was classified based on the calculation results.

The data used in the research were derived from the statistical yearbook published by the Dalian Municipal Bureau of Statistics [26] and the health monitoring point of the Dalian Municipal Health Bureau [27]. As the most authoritative and official data released by the Chinese government, researchers and government departments have been using this data to study medical developments in China. The Dalian Health Monitoring Point data was initially a two-level monitoring system consisting of administrative districts and streets, with a total of 141 monitoring points. In 2008, with the launch of the medical reform policy pilot, the Dalian Health Monitoring Point evolved into a three-level monitoring system consisting of administrative district-, street-, and community-levels, and the number of monitoring points increased to 1266. The city’s core area, composed of Zhongshan, Xigang, and Shahekou Districts, saw an increase of about 4.6 million in the number of residents (inhabited population), which accounted for 78.3% of city’s total population. To ensure the accuracy and stability of the research data, we selected the 2008–2017 statistical yearbook (with 1266 monitoring points), since the health care reform policy was initiated in 2008. The statistical yearbook and the health monitoring point data consists of a three-level statistical system where the information is collected and aggregated using a step-by-step review and reporting the procedure to ensure the integrity and accuracy of statistical information. If any statistical item changes significantly or any inaccuracy is found, a repeat in analytical work would be conducted to ensure the accuracy of the data.

### 2.2. Procedure

We divided the regional medical evaluation system into three criteria layers, consisting of the social life system (*A1*), economic system (*A2*), and medical system (*A3*). The social life system (*A1*) includes population pressure (*B1*) and the cost of living (*B2*). The economic system (*A2*) includes regional economy (*B3*) and government medical input (*B4*). The medical system (*A3*) includes basic medical facilities (*B5*), medical staffing (*B6*), and resident medical insurance (*B7*). We further subdivided the second criterion layer. Population pressure (*B1*) includes population size (*C1*), population density (*C2*), population mechanical change rate (*C3*), population over 60 (*C4*), and rural population (*C5*). Cost of living (*B2*) includes per capita disposable income (*C6*), housing prices (*C7*), and health care consumption (*C8*). Regional economy (*B3*) includes regional gross domestic product (GDP) (*C9*). Government medical input (*B4*) includes government health care expenditure (*C10*). Primary medical facility (*B5*) contains medical bed count (*C11*), and the number of medical institutions (*C12*). Medical staffing (*B6*) includes the number of doctors and nurses (*C13*), and the number of practicing and assistant doctors (*C14*). Residents’ medical security (B7) includes the number of residents insured (*C15*), the number of doctors per thousand (*C16*), and the number of nurses per thousand (*C17*). In this study, the data from islands, such as the Changhai County with a population less than 5000, have been excluded in the analysis (Table 1).

### 2.3. Weighted TOPSIS Model

#### Standardization of Indicators

The existing indicator data needed to be standardized before determining the index weights. The standardization method should be applied to both weight determination methods at the same time to avoid the influence of different index dimensions on the standardized results. In this study, we used the following standardized methods:

(1) $$y_{ij}=\frac{x_{ij}}{\sum_{i=1}^{m}x_{ij}},i=1,2,3\ldots m;j=1,2,3,\ldots n$$

where $x_{ij}$ is the j-th index value in the i-th study area in the matrix composed of the obtained m research areas and *n* evaluation indexes. Using this method, the normalized matrix $Y\;=\;\left(y_{ij}\right)m\;\times \;n$ can be obtained.

### 2.4. Analytic Hierarchy Process

Indicator weight reflects the different degrees of indicators in the evaluation process and provides the relative importance of indicators in the decision-making (or evaluation) process. The concept of AHP combines quantitative and qualitative analysis with a systematic and hierarchical modeling approach. Here, we used AHP to obtain the subjective weight of each indicator. We used the Yaahp software to check the consistency of each indicator. The test result was 0.0624 (<1), which is of research significance. We drew each indicator layer according to the regional medical evaluation system and used the automatic adjustment algorithm function to calculate the index of each indicator. When assigning subjective weights, we developed the indicator system using previous studies [28,29] and combined the software calculation results to obtain the subjective weight of each indicator.

### 2.5. Entropy Weight Method

The entropy weight method (EWM) is a comprehensive evaluation technique that can be used for multiple objects and indicators. The evaluation results were based mainly on objective data and were mostly unaffected by subjective factors. In this study, the entropy weight method was used to determine the objective weight by the magnitude of the index variability, which largely avoided bias from human interference. The following equations were used:

(2) $$y_{ij}\;=\;\frac{u_{ij}}{\sum_{i\;=\;1}^{m}u_{ij}}$$

(3) $$E_{j}\;=\;-\frac{1}{\ln m}\overset{m}{\underset{i\;=\;1}{\sum }}y_{ij}{\ln y}_{ij}$$

where $u_{ij}$ is the labeled value of the i-th scheme under the j-th index in the normalized matrix; $y_{ij}$ is the proportion of the labeled value of the i-th scheme under the j-th index in the standardized matrix; and, $E_{j}$ represents the information entropy of the j-th index in the evaluation matrix Y. When the amount of information contained in a particular indicator is consistent with all the research areas, the information entropy of the indicator reaches the maximum value, where $E_{j}\;=\;1$.

Before performing entropy weight calculations, the information utility value of the indicator should first be calculated. The value of the utility value $D_{j}$ of the indicator, information is dependent on the indicator’s information entropy $E_{j}$, which is then used to determine the size of the indicator’s entropy weight $w_{j}^{\prime \prime }$. The higher the utility information value of an indicator, the greater its weight. The following equations were used to determine the indictor’s entropy weight:

(4) $$D_{j}\;=\;1\;-\;E_{j}$$

(5) $$w_{j}^{\prime \prime }\;=\;\frac{D_{j}}{\sum_{j\;=\;1}^{n}D_{j}}$$

### 2.6. Comprehensive Weight Model

To overcome the subjectivity of the indicator evaluation by experts and decision-makers in AHP, and the entropy weight method, objective evaluation of each index was carried out. The overall weight $w_{j}$ of each index was calculated using the equation:

(6) $$w_{j}{\;=\;\alpha w}_{j}^{\prime }{\;+\;(1\;-\;\alpha )w}_{j}^{\prime \prime }$$

In this study, the value of α was set to 0.5, as recommended by previous research. Using proportional changes in the subjective and objective weights, Ma, J. et al. [30] employed sensitivity analysis and found that α equal to 0.5 is relatively reasonable (Table 2).

### 2.7. TOPSIS Model

TOPSIS (Technique for order preference by similarity to ideal solution) is a commonly used method for multi-objective decision analysis, which can be used in multiple applications. This method is characterized by having no special requirements on data, good flexibility and simplicity, and is applicable for a variety of uses. Given its flexibility, TOPSIS is a widely used evaluation method in the fields of tourism and medicine (e.g., [31,32,33]). The calculations for the TOPSIS model are based on the normalized original index data matrix. The cosine method is used to find the best and worst values in the limited scheme, which are then used as the basis for evaluation. Before using the TOPSIS model, the evaluation indicators have to be standardized first and are divided into high-quality and low-quality indicators. High-quality indicators are the parameters that have greater effect on promoting the development of the healthcare industry. To facilitate the elimination of the dimensional constraints among the indicator data in the evaluation index matrix X (${X\;=\;(x}_{ij})\;m\;\times \;n$), we used the reciprocal method (1/X × 100) to convert all low-quality indicators into high-quality indicators. The converted indicator was calculated using the standardized formula:

(7) $$Z_{ij}=\{\begin{array}{l} \frac{x_{ij}}{\sqrt{\sum_{i\;=\;1}^{n}{\left(x_{ij}\right)}^{2}}}\;(\mathrm{Original}\;\mathrm{high}-\mathrm{quality}\;\mathrm{indicator})\\ \frac{x_{ij}^{\prime }}{\sqrt{\sum_{i\;=\;1}^{n}{\left(x_{ij}^{\prime }\right)}^{2}}}\;(\mathrm{Original}\;\mathrm{low}-\mathrm{priority}\;\mathrm{index}) \end{array}$$

where $Z_{ij}$ is the standardized indicator value; $x_{ij}$ is the high-quality indicator; and $x_{ij}^{\prime }$ is the low-quality indicator. The high-quality indicators in the medical evaluation system proposed in this study are government health care expenditure (*C10*), the number of doctors and nurses (*C13*), medical bed count (*C11*), the number of medical institutions (*C12*), total number of residents with medical insurance (*C15*), the number of doctors per thousand residents (*C16*), the number of nurses per thousand residents(*C17*), number of licensed doctors and assistant doctors (*C14*), disposable income per capita (*C6*), and regional GDP (*C9*). The low-quality indicators include health care consumption (*C8*), population size (*C1*), population density (*C2*), population machinery change rate (*C3*), population over 60 (*C4*), rural population (*C5*), and house prices (*C7*).

We then have to determine the optimal solution X^+^ and the worst solution X^−^: The optimal solution X^+^ consists of the maximum value in each column of the matrix X: X^+^= (maxZ_1_, maxZ_2_, …, maxZ*_m_*), (*n* = 1, 2…, *n*). The worst-case X^−^ consists of the minimum value in each column of the matrix X: X^−^ = (minZ_1_, minZ_2_, …, minZ*_m_*), (*n* = 1, 2, …, *n*).

The distance $D_{i}^{+}$ and $D_{i}^{-}$ of each evaluation indicator was calculated from X^+^ and X^−^:

(8) $$D_{i}^{+}\;=\;\sqrt{\overset{m}{\underset{i\;=\;1}{\sum }}w_{j}{\left({\mathrm{maxZ}}_{ij}{\;-\;Z}_{ij}\right)}^{2}},\;(i\;=\;1,2,\;\ldots ,n)$$

(9) $$D_{i}^{-}\;=\;\sqrt{\overset{m}{\underset{i\;=\;1}{\sum }}w_{j}{\left({\mathrm{minZ}}_{ij}{\;-\;Z}_{ij}\right)}^{2}},\;(i\;=\;1,2,\;\ldots ,n)$$

where $D_{i}^{+}$ is the proximity level of the evaluation vector to the optimal value, and $D_{i}^{-}$ is the proximity level of the evaluation vector to the least ideal value. The proximity of each evaluation index to the optimal solution X^+^ ($C_{i}$) was then calculated using the equation:

(10) $$C_{i}=\frac{D_{i}^{-}}{D_{i}^{+}{\;+\;D}_{i}^{-}},\;i\;=\;1,2,\;\ldots ,n$$

where $C_{i}$ ranges from 0 to 1. When the $C_{i}$ value of a scheme is closer to 1, it means that the scheme is closer to the optimal value. When $C_{i}\;=\;0$ or $C_{i}\;=\;1$, it means that the solution value is the least ideal solution or the optimal solution.

Obstacle model

To explore the factors that constrain healthcare developments in each region, we used the obstacle model to gauge the standardized data. The obstacle model calculates the degree of obstruction for each indicator and was calculated as follows:

(11) $$O_{ij}{\;=\;1\;-\;x}_{ij}{,\;I}_{j}=\frac{O_{ij}{\cdot w}_{j}}{\sum_{j\;=\;1}^{n}O_{ij}{\cdot w}_{j}}$$

where $O_{ij}$ is the deviation degree of the index and is equal to the difference between the single target and the system development goal; $w_{j}$ is the contribution degree of the single index factor to the system, which is represented by the overall weight; and, $I_{j}$ is the obstacle degree of the single indicator.

## 3. Results

### 3.1. TOPSIS Scores of Urban and Rural Medical Care in Dalian

**Figure 1:**
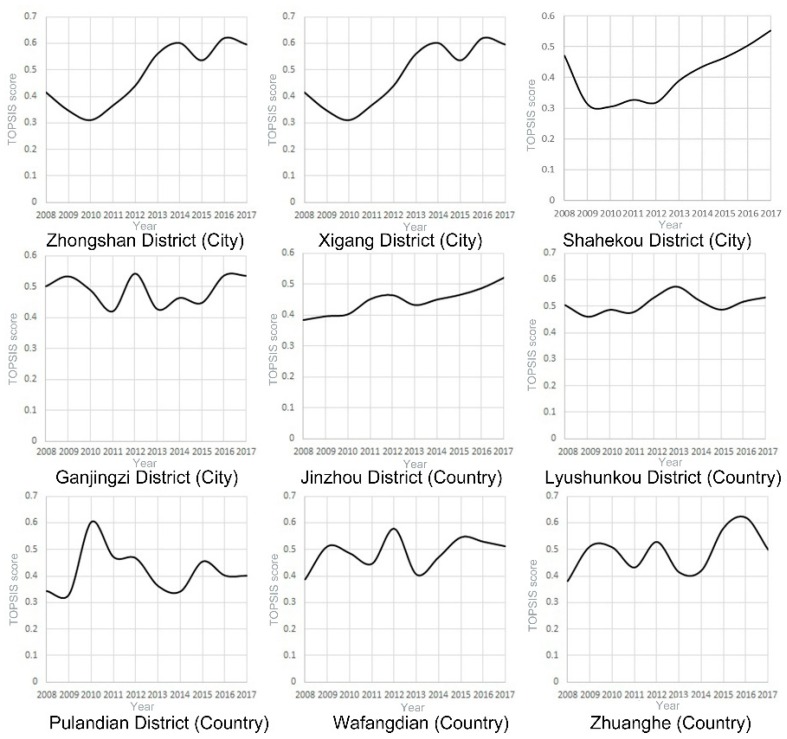
2007–2018, Dalian urban and rural technique for order preference by similarity to ideal solution (TOPSIS) curve.

**Figure 2:**
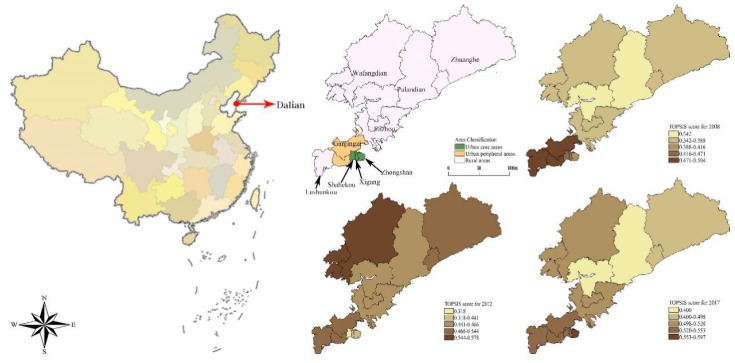
2008–2017 Dalian City, urban and rural medical TOPSIS score change.

Score curves for each region are used to visualize the healthcare changes in each region. Maps for 2008, 2012, and 2017 are presented to show the changes in the urban–rural gap through geospatial concepts. From 2008 to 2017, the scores of medical services in various regions of Dalian increased from 0.3419‒0.5037 to 0.4002‒0.5967 (Table 3). When examining the score curves of each region over the years (as shown in Figure 1 and Figure 2), the healthcare services in the city’s core areas (Zhongshan, Xigang, and Shahekou Districts) have continued to rise since 2010. Although the scores in Zhongshan and Xigang Districts had decreased in 2015 by 10.9% and 18.3%, respectively, this decline did not have a substantial impact on the development of medical services in these two regions. Healthcare developments in the urban fringe district of Ganjingzi and in the rural areas (i.e., Lushunkou, Jinzhou, and Pulandian Districts, Wafangdian City, and Zhuanghe City) showed a fluctuating trend. Areas with the most substantial fluctuations include Ganjingzi District, Pulandian District, Wafangdian City, and Zhuanghe City. The rural areas of Pulandian District (76.2%) and Zhuanghe City (49.5%) had the highest annual rate of change, while the vicinities with relatively stable scores were Jinzhou District (3.1%) and Lushunkou District (3.2%).

In the urbanized part of the city, Ganjingzi District had the highest score at 0.498 in 2008. But its score began to decrease after 2009, while the scores in the other urban areas continued to rise from 2010. The highest score in rural areas in 2008 was Lushunkou District (0.491), while the lowest was Jinzhou District (0.420). By 2017, Zhongshan District replaced Ganjingzi District as having the highest regional average score (0.584) among the urban areas. Lushunkou District (0.527) remained the highest score among the rural districts, while the Pulandian District became the worst-scoring area (0.391). The average scores for Zhuanghe City (0.507) and Jinzhou District (0.471) increased significantly from 2008 to 2012.

### 3.2. Indicator Contribution

The indicator contribution scores were calculated for each region, and the summary is presented in Table 1. For analysis, we selected the top four indicators for each region (see Table 4) and highlighted the indicators that had at least 5% contributions. For urban core areas, per capita disposable income (C6, 8.56–8.63%), regional GDP (C9, 7.78–8.54%), government healthcare expenditure (C10, 7.12–8.2%), and house prices (C7, 5.91–7.04%), are the top four indicators (where C6 > C9 > C10 > C7). For peripheral urban areas (Ganjingzi), number of medical institutions (C12, 10.29%), per capita disposable income (C6, 9.83%), government health care expenditure (C10, 9.09%), and regional GDP (C9, 8.71%) were the top four indicators (where C12 > C6 > C10 > C9). For the rural areas, the top four indicators were number of medical institutions (C12, 10.68–2.08%), per capita disposable income (C6, 10.50–10.85%), government health care expenditure (C10, 9.01–9.22%), and regional GDP (C9, 8.06–8.60%), similar to the ranking in the urban peripheral region (where C12 > C6 > C10 > C9). The results indicate that increasing per capita disposable income in the urban core region is the most effective way of achieving healthcare improvements, while in the urban fringe and rural areas, building more medical institutions is the most efficient means to improve the healthcare system.

**Figure 3:**
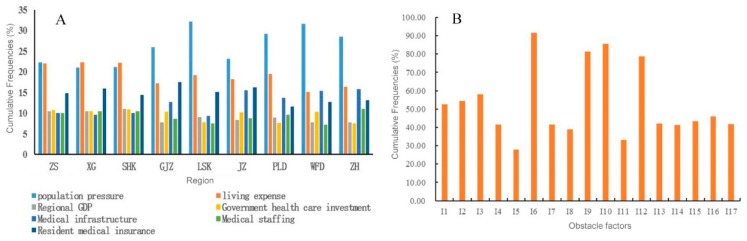
Frequency of obstacles and cumulative frequency histogram of obstacles in various regions. (**A**) Contribution of indicators by region; (**B**) Cumulative frequency of index obstacle degree.

We calculated the cumulative frequency of the second criterion layer (B1–B7) for each region and visualized them in a histogram (Figure 3A). For both urban and rural areas, population pressure (B1, 21–32.1%) and cost of living (B2, 15.1–22.3%) are the major obstacles to healthcare service. For urban core areas, population pressure (B1, 21–25.9%), cost of living (B2, 17.2–22.3%), and resident medical insurance (B7, 14.4–17.5%) are the main impediments. For the rural areas, four indicators presented the most obvious impact healthcare development: population pressure (B1, 23.1–31.6%), cost of living (B2, 15.1–19.4%), medical infrastructure (B5, 13.7–15.8%), and residential health care (B7, 11.6–16.2%).

### 3.3. Obstructing Urban and Rural Medical Development Indicators

To visualize the factors inhibiting healthcare progress in Dalian, we generated the cumulative frequency histogram (see Figure 3B) of indicators at the third criterion layer (C1–C17). Based on the cumulative frequency of obstacles, the top four indicators included per capita disposable income (91.5%), regional GDP (81.3%), government health care expenditure (85.5%), and the number of medical institutions (78.7%). In addition, indicators with a cumulative frequency greater than 50% also included population mechanical change rate (58.1%), population density (54.3%), and population (52.6%). The results suggest that if the government aims to efficiently address healthcare reforms, it must not only address the local economic conditions and financial costs of health services but also consider the impact of the population in its policies and reforms.

To narrow down the number of indicators to be analyzed, we limited our focus to indicators with obstacles greater than 10% (Table 5). For urban core areas, per capita disposable income (I6, 8.7–11.3%), regional GDP (I9, 7.8–11%), and government health care expenditure (I10, 10.3–10.9%) were found to be the indicators having the most significant obstacles for healthcare development (I6 > I9 > I10). For peripheral urban areas (Ganjingzi District), the leading indicators hindering healthcare were the number of medical institutions (I12, 10.5%), and government health care expenditure (I10, 10.3%). For rural areas, the indicators impeding progress in healthcare services varied. For Lyshunkou District, it was per capita disposable income (I6, 10.2%). For Jinzhou District and Wafangdian City, they were healthcare expenditures (I10, 10.1%–10.3%) and the number of medical institutions (I12, 13%,–12.3%). And for Pulandian District and Zhuanghe City, the indicators were per capita disposable income (I6, 10.1%,–11.4%) and the number of medical institutions (I12, 11%,–11.9%).

**Figure 4:**
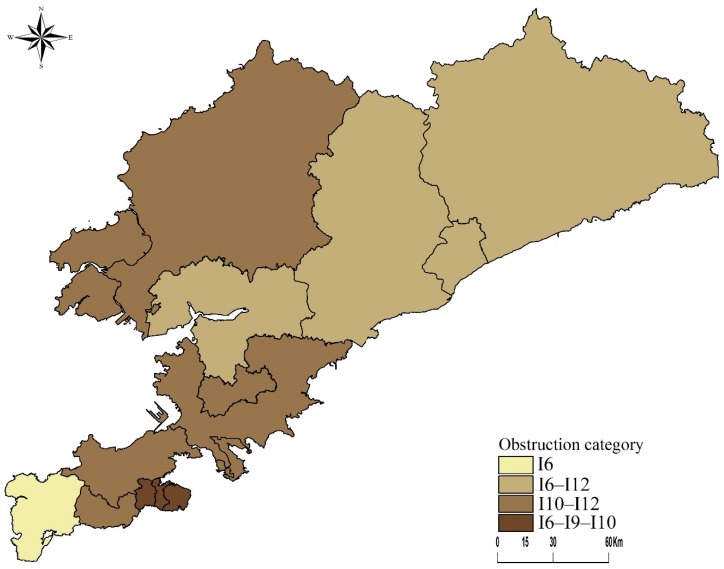
Different types of obstacles in medical undertakings by region.

We used the local spatial clustering function in ArcGIS10.5 to perform local spatial cluster analysis on the obstacle data (I1–I17) for each region and classified them according to the type of obstacle (see Figure 4). According to the type of resistance, the regions can be categorized into four groups: unipartite resistance mode (I6), bipartite resistance mode (I6-I12 and I10-I12), and tripartite resistance mode (I6-I9-I10). The area affected by unipartite resistance mode (I6) was the Lushunkou District. The areas covered by bipartite resistance mode (I6-I12) were Pulandian District and Zhuanghe City. The areas affected by bipartite resistance mode (I10-I12) included Ganjingzi District, Jinzhou District, and Wafangdian City. The areas influenced by tripartite resistance mode (I6-I9-I10) were Zhongshan District, Xigang District, and Shahekou District.

## 4. Discussion

After using the weighted TOPSIS model and the obstacle model to analyze the urban–rural gap in Dalian and identify the significant variables affecting its healthcare developments, we found three main findings.

### 4.1. Health Services in Urban vs. Rural Areas

The health services in the urban districts are developing well, but the progress in healthcare in rural areas remains unstable. According to the results of the weighted TOPSIS calculation, the score range of urban areas increased from 0.416–0.502 in 2008 to 0.537–0.597 in 2017, while the scores in rural areas increased from 0.342–0.503 in 2008 to 0.400–0.534 in 2017. At first glance, the overall quality of healthcare services in both urban and rural areas has shown significant improvements. However, the change in healthcare quality in rural areas was more pronounced and unstable, while the general trend in urban areas was more regular and stead. Consider the rural district of Pulandian as an example. In 2008, the regional score was 0.342. In 2010, it was 0.602. And in 2017, it was 0.400. The explanation behind the instability in healthcare development in rural areas is multifaceted. Population mobility, economic development, and employment rate can have a significant impact on the progress of healthcare systems [34,35].

By comparing the data of various indicators in the urban and rural areas of Dalian from 2008 to 2017, we found that the improvements of medical services in rural areas were more susceptible to changes from population density, government healthcare expenditures, and per capita disposable income, as compared to the urban communities. In 2017, the average population density in Dalian’s rural areas (537 people/km^2^) was much lower compared to its urban districts (6366 people/km^2^), but the extent of the urban spaces was only 4.32% of the rural regions. Compared with densely populated urban areas, upgrading and improvements of healthcare services in rural districts would require more resources and entail more comprehensive planning to reach remote communities. In China, the development of the urban healthcare system is supported by national funds and local revenue. In recent years, the Chinese government has given more and more attention to rural communities. To assist in improving the healthcare system, the Chinese government has provided plenty of policy support for medical services in rural communities [36,37,38]. The average government healthcare expenditure in rural areas (378 million yuan ≈ 56.42 million dollars) has become much higher compared with the urban districts (143 million yuan ≈ 21.34 million dollars). Consequently, the growth of the healthcare system in rural areas has become mainly dependent on the government’s financial support. 

### 4.2. Factors Promoting Healthcare Development

While some factors promoting healthcare development in urban areas were similar to those in the rural regions, other parameters were specific only to urban or rural areas. Through the analysis of indicator contributions, we found that for both urban and rural areas, regional GDP, per capita disposable income, and government healthcare expenditures significantly influence improvements in health services. Therefore, in improving urban and rural healthcare systems, the Chinese government has a leading role to play. Apart from supporting the development of the local economy, the government can invest more in healthcare spending, which will have significant effects in directly enhancing health services in the urban and rural areas. The analysis of the indictor contributions shows that in urban and rural areas, regional GDP and government healthcare expenditures had a significant role in promoting improvements in medical services. The Chinese government should strengthen its role in supporting the development of urban and rural medical services. By developing the local economy, more income can be used to reduce the disparity between urban and rural medical care. 

**Figure 5:**
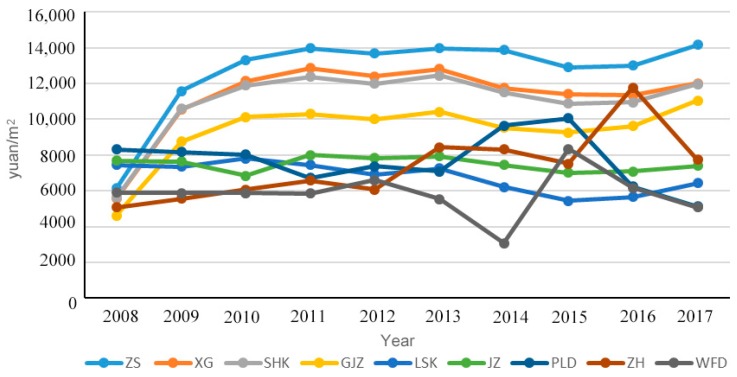
House prices in various regions of Dalian, 2008–2017.

In terms of house prices, the average home values in urban and rural areas for 2008–2017 were 11,078 yuan/m^2^ (1653.4 dollars/m^2^) and 7019 yuan/m^2^ (1047.6 dollars/m^2^), respectively. House prices in urban areas were significantly higher. In the urban districts (see Figure 5, the average house value (11,650.3 yuan/m^2^ ≈ 1738.9 dollars/m^2^) was about 1.7 times greater than in the rural areas, which means urban residents spend more in purchasing their homes. Based on geography’s location theory [39], residents of urban areas will need less time to reach medical facilities and enjoy greater convenience and access to high-quality medical services; however, they will also bear higher economic costs [40,41]. Residents in rural communities, who live farther from urban centers, will see their cost of living and land value significantly reduced; but this comes with the price of having limited access to services, including high-quality medical care. For most rural areas, the deficiency in the number of medical institutions was clearly evident. In Dalian, there were a total of 12 tertiary hospitals, but only two were found in the rural regions. And although the number of hospitals was greater in rural communities (98) than in the urban districts (60), given the size differential between the rural and urban regions, the density of secondary and tertiary hospitals in cities (19.7/km^2^) was significantly higher than in rural communities (1.1/km^2^). The lack of high-level medical institutions in rural areas will prevent residents in the area from receiving better medical services.

### 4.3. Obstacles to Healthcare Improvements

Obstacles hindering healthcare improvements varied throughout the region. Based on the results of the obstacle model, the top four rankings for the cumulative frequency of index obstacles in each region were per capita disposable income, regional GDP, government healthcare expenditure, and the number of medical institutions. However, these indicators varied distinctly when analyzing at the district level. We found that there were more significant obstacles for healthcare progress in the core region than in the urban peripheral and rural areas. While the core region enjoyed a stronger economy and more developed infrastructure compared to the rural districts, the complexity of the urban network [42,43,44] led to the vulnerabilities and fragility in the urban healthcare system [45,46]. When a variety of factors contributed to any given operation, such as the healthcare system, confusion may easily occur. Progress in health services in the core areas should, therefore, follow the principle of coordinated development. In the urban peripheral and rural areas, the factors that impede improvements in the healthcare system were more diverse. For areas near the city’s core, per capita disposable income, which can directly be connected with the population’s economic conditions, was a significant obstacle for healthcare progress. With districts located farther from the core area, population density, and the number of medical institutions became significant impediments in the expansion of health services. Thus, it is essential to understand the dynamics between population density and the number of medical institutions, particularly in rural communities, to provide sufficient and quality medical services to rural residents.

### 4.4. Application of this Research Model in Other Fields

We introduced comprehensive weights into the TOPSIS model and the obstacle degree model in the calculations, to avoid errors caused by using only either subjective weights or objective weights. Since the TOPSIS model is a multi-objective decision model, it is suitable for this type of research. The use of the obstacle degree model allows the determination of significant constraints in the index system. The approach developed in this study would be applicable in evaluating the healthcare industry and other applications, such as public service facility evaluation, regional development, and natural resource risk management. For example, when assessing water resource risk management, the TOPSIS model can be used to evaluate various indicators, while the obstacle degree model can be utilized in determining the constraints.

### 4.5. Limitations of the Study

There may be some possible limitations in this study that could be improved in future research. First, our primary approach established a regional medical evaluation system using a number of quantitative indicators. However, in reality, many other factors could affect the development of the healthcare system, such as the people’s differential preferences (e.g., in choosing their hospitals), the efficiency of medical diagnosis and treatment, and the survival and cure rates. For future research, these and other indicators can be added to the assessment.

Second, although we have identified indicators that promote and/or hinder progress in health services, we did not assess the intensity and extent to which these factors impact the healthcare system. Future studies can include quantifying the degree of indicator impact and focus on the internal mechanisms of parameters to have a more comprehensive evaluation system. Third, in this study, we employed the entropy and AHP methods to calculate the subjective and objective weights of each index, which were then used to obtain the overall weights. However, the values in the comprehensive weight model may not be applicable to other regions or to other time frames. In addition, the research area, Dalian city, while being a typical large city in China, cannot be considered as representative of all regions. Other cities, though seemingly comparable in features, may yield different results due to specific local aspects.

Finally, none of the parameters we used were spatial in nature, which limited the extent of indicators in this study. Furthermore, our use of the ArcGIS software for local spatial clustering, while being very efficient, is highly dependent on parameters, which may cause clustering results to deviate from actuality. 

## 5. Conclusions

In this study, we established a regional medical evaluation index system based on a modified TOPSIS model, which used subjective and objective weights calculated from AHP and EWM to evaluate healthcare developments in urban and rural areas. In analyzing the medical care in the urban and rural districts of Dalian from 2008 to 2017, we examined the indicators and variables that promote and hinder the development of medical services. Based on our results, the following conclusions were drawn:

- Based on the results of the weighted TOPSIS model and the obstacle model, the healthcare services in Dalian’s urban areas have steadily increased, while those in the rural regions have been unstable and erratic. Although the urban–rural healthcare disparity has generally narrowed, continued progress is not assured.
- Based on the location theory, residents in urban areas are more influenced by economic factors, while those in rural areas are more affected by time considerations. Therefore, when promoting the development of healthcare services in urban areas, policies and measures should consider the impact of land prices and per capita disposable income. For rural areas, constructing more medical institutions can effectively reduce the impact of time costs on the progress of health services.
- Different factors may hinder the development of healthcare systems in urban and rural areas. Urban areas should focus on coordinated development to address impediments in healthcare progress, while rural areas should address healthcare concerns based on local needs and conditions.

## Figures and Tables

**Table 1:** Regional medical evaluation system.

Primary Index	Secondary Index	Tertiary Index	Average Value	Standardized Mean
social life system (*A1*)	population pressure (*B1*) (We defined population pressure as a variety of population data that can affect urban development within the city.)	population size (*C1*)	5,831,091 people	0.3162
		population density (*C2*)	498,412.1 people/km^2^	0.3162
		population mechanical change rate (*C3*)	3.09%	0.2475
		population over 60 (*C4*)	498,412 people	0.3052
		rural population (*C5*)	2.276 million people	0.3129
	the cost of living (*B2*) (We defined the cost of living as income and expenditure necessary for urban living.)	per-capita disposable income (*C6*)	37,282.2 yuan	0.3103
		housing prices (*C7*)	8823.21 yuan/m^2^	0.3146
		health care consumption (*C8*)	35,552.15 yuan	0.2957
economic system (*A2*)	regional economy (*B3*) (We used regional GDP as an indicator of the regional economy.)	regional GDP (*C9*)	16.197 billion yuan	0.3100
	government medical input (*B4*) (We used government expenditure on health as an indicator of medical construction.)	government health care expenditure (*C10*)	437.9973 million yuan	0.3005
medical system (*A3*).	primary medical facility (*B5*) (We used primary medical facility. The institution was defined as various types of hospitals that can meet the medical needs of urban residents, and the total size was represented by the number of medical beds.)	medical bed count (*C11*)	35,552.15 beds	0.3126
		the number of medical institutions (*C12*)	1619.37 institutions	0.3077
	medical staffing (*B6*) (To fully display the scale of medical personnel in Dalian, we have counted the number of doctors and in-service doctors in all medical institutions’ number of nurses.)	the number of doctors and nurses (*C13*)	37282 people	0.3071
		the number of practicing and assistant doctors (*C14*)	8800.28 people	0.2470
	resident medical insurance (*B7*) (We took the number of people covered by medical insurance as one of the indicators reflecting residents medical security, but there were still residents who did not participate in medical insurance. To balance the supply and demand relationship, we added the number of doctors per 1000 people. with the number of nurses per thousand people to avoid research errors.)	the number of residents insured (*C15*)	342,313 people	0.2822
		the number of doctors per thousand (*C16*)	3.56 people	0.31401
		the number of nurses per thousand (*C17*)	7.14 people	0.3118

**Table 2:** Analytic hierarchy process (AHP) weight, entropy weight, and comprehensive weight of each index.

Index	EWM	AHP	Comprehensive Weight
C1	0.047	0.069	0.058
C2	0.049	0.042	0.0455
C3	0.029	0.042	0.0355
C4	0.056	0.069	0.0625
C5	0.086	0.026	0.056
C6	0.025	0.059	0.042
C7	0.108	0.034	0.071
C8	0.044	0.041	0.0425
C9	0.043	0.039	0.041
C10	0.075	0.039	0.057
C11	0.049	0.135	0.092
C12	0.035	0.112	0.0735
C13	0.071	0.138	0.1045
C14	0.121	0.049	0.085
C15	0.057	0.019	0.038
C16	0.053	0.039	0.046
C17	0.052	0.048	0.05

EWM—entropy weight method; AHP—analytic hierarchy process.

**Table 3:** Technique for order preference by similarity to ideal solution (TOPSIS) scores in urban and rural areas, 2008-2017.

Year	Urban				Rural				
	ZS	XG	SHK	GJZ	JZ	LSK	PLD	WFD	ZH
2008	0.4164	0.4662	0.4712	0.5023	0.3850	0.5037	0.3419	0.3876	0.3808
2009	0.3480	0.3127	0.3137	0.5346	0.3970	0.4591	0.3291	0.5118	0.5120
2010	0.3116	0.3146	0.3048	0.4897	0.4035	0.4856	0.6021	0.4859	0.5081
2011	0.3665	0.3485	0.3272	0.4208	0.4514	0.4747	0.4701	0.4465	0.4323
2012	0.4407	0.4235	0.3181	0.5436	0.4641	0.5337	0.4657	0.5778	0.5287
2013	0.5608	0.5107	0.3894	0.4275	0.4326	0.5752	0.3605	0.4056	0.4141
2014	0.6022	0.5725	0.4344	0.4646	0.4503	0.5225	0.3404	0.4719	0.421
2015	0.5368	0.4678	0.4646	0.4484	0.4647	0.4862	0.4538	0.546	0.582
2016	0.6208	0.5611	0.5037	0.5378	0.4865	0.5173	0.4003	0.5289	0.619
2017	0.5967	0.5466	0.5525	0.5365	0.5198	0.5337	0.4002	0.5116	0.4982

ZS—Zhongshan district; XG—Xigang district; SHK—Shahekou district; GJZ—Ganjingzi district; JZ—Jinzhou district; LSK—Lyushunkou district; PLD—Pulandian district; WFD—Wafangdian district; ZH—Zhuandhe district.

**Table 4:** Contribution of various indicators to medical undertakings.

Indicator	ZS	XG	SHK	GJZ	LSK	JZ	PLD	WFD	ZH
C1	**6.04%**	**5.70%**	**5.57%**	**5.91%**	**5.56%**	**5.98%**	**5.73%**	**8.53%**	**5.20%**
	5th	6th	7th	6th	8th	6th	6th	4th	9th
C2	**5.83%**	**5.69%**	**5.85%**	**5.91%**	3.87%	**5.95%**	**10.39%**	4.83%	**5.29%**
	6th	7th	6th	7th	16th	7th	3rd	10th	7th
C3	4.37%	4.27%	4.23%	**5.07%**	**5.66%**	**5.16%**	4.66%	**5.60%**	**5.91%**
	10th	12th	12th	10th	6th	9th	11th	7th	6th
C4	**5.18%**	**6.20%**	**7.36%**	3.94%	4.63%	3.08%	3.38%	**6.33%**	4.95%
	7th	4th	4th	15th	12th	16th	15th	6th	12th
C5	-	-	-	**5.52%**	**5.11%**	3.82%	4.66%	4.79%	**5.04%**
				8th	9th	15th	12th	11th	10th
C6	**8.57%**	**8.63%**	**8.56%**	**9.83%**	**10.35%**	**10.37%**	**10.52%**	**10.85%**	**10.50%**
	1st	1st	1st	2nd	2nd	2nd	2nd	2nd	2nd
C7	**7.04%**	**5.91%**	**5.97%**	**5.19%**	4.84%	4.32%	3.82%	2.99%	2.92%
	4th	5th	5th	9th	11th	14th	13th	17th	17th
C8	4.11%	4.17%	4.75%	3.81%	4.50%	4.80%	4.67%	4.11%	3.78%
	13th	13th	9th	16th	13th	10th	10th	13th	16th
C9	**8.54%**	**8.22%**	**7.77%**	**8.71%**	**8.11%**	**8.60%**	**8.06%**	**8.46%**	**8.47%**
	2nd	2nd	2nd	4th	4th	4th	5th	5th	4th
C10	**8.20%**	**7.12%**	**7.64%**	**9.09%**	**9.34%**	**9.67%**	**9.38%**	**9.22%**	**9.01%**
	3rd	3rd	3rd	3rd	3rd	3rd	4th	3rd	3rd
C11	3.16%	2.75%	2.47%	2.39%	5.66%	2.66%	2.92%	3.09%	4.26%
	16th	16th	16th	17th	7th	17th	17th	16th	15th
C12	4.48%	**5.69%**	**5.40%**	**10.29%**	**11.65%**	**12.01%**	**11.63%**	**12.08%**	**10.68%**
	8th	8th	8th	1st	1st	1st	1st	1st	1st
C13	4.47%	4.49%	4.56%	4.87%	4.04%	4.64%	**5.09%**	4.35%	5.21%
	9th	10th	11th	12th	15th	12th	9th	12th	8th
C14	3.19%	3.62%	3.66%	4.13%	3.66%	4.43%	**5.17%**	3.40%	**6.93%**
	15th	14th	13th	14th	17th	13th	8th	15th	5th
C15	4.25%	4.36%	4.75%	4.97%	4.97%	4.66%	**5.33%**	4.90%	4.79%
	11th	11th	10th	11th	10th	11th	7th	9th	13th
C16	4.18%	4.55%	3.60%	**8.15%**	**6.08%**	**5.51%**	3.72%	**5.26%**	4.98%
	12th	9th	14th	5th	5th	8th	14th	8th	11th
C17	3.34%	3.11%	2.91%	4.64%	4.37%	**6.74%**	3.27%	3.61%	4.48%
	14th	15th	15th	13th	14th	5th	16th	14th	14th

ZS—Zhongshan district; XG—Xigang district; SHK—Shahekou district; GJZ—Ganjingzi district; JZ—Jinzhou district; LSK—Lyushunkou district; PLD—Pulandian district; WFD—Wafangdian district; ZH—Zhuandhe district. Variables in bold letters indicate contributions greater than 5%.

**Table 5:** Factor of each area.

Obstacle Factor	Summary of Indicator Information	ZS	XG	SHK	GJZ	LSK	JZ	PLD	WFD	ZH
I1	population size	5.38	5.07	4.94	5.56	8.28	5.62	5.28	7.74	4.73
I2	population density	5.98	5.06	5.19	5.55	8.15	5.59	9.58	4.42	4.81
I3	population mechanical change rate	5.15	5.16	5.44	5.16	5.98	5.28	6.85	9.25	9.76
I4	population over 60	5.71	5.67	5.61	3.74	4.48	2.94	3.17	5.79	4.56
I5	rural population	-	-	-	5.88	5.24	3.64	4.33	4.35	4.59
I6	per capita disposable income	10.83	11.22	11.25	8.66	10.20	9.38	11.35	8.49	10.12
I7	housing prices	6.33	6.30	6.32	4.90	4.64	4.06	3.55	2.74	2.70
I8	health care consumption	4.80	4.81	4.53	3.67	4.38	4.74	4.53	3.90	3.53
I9	regional GDP	10.75	10.44	10.99	7.76	9.05	8.31	8.83	7.79	7.81
I10	government health care expenditure	10.38	10.37	10.88	10.26	7.76	10.10	7.60	10.32	7.40
I11	medical bed count	4.85	4.45	4.21	2.24	5.54	2.52	2.72	2.86	3.89
I12	the number of medical institutions	5.12	5.15	5.86	10.48	3.77	12.96	11.00	12.45	11.94
I13	the number of doctors and nurses	5.11	5.12	5.17	4.65	3.94	4.45	4.82	4.05	4.74
I14	the number of practicing and assistant doctors	4.86	5.24	5.25	3.95	3.53	4.21	4.78	3.10	6.31
I15	the number of residents insured	5.00	5.12	4.53	4.94	4.91	4.54	5.15	4.60	4.48
I16	the number of doctors per thousand	4.73	5.05	4.22	8.22	5.88	5.24	3.43	4.79	4.53
I17	the number of nurses per thousand	5.01	5.77	5.60	4.36	4.25	6.42	3.02	3.34	4.11

ZS—Zhongshan district; XG—Xigang district; SHK—Shahekou district; GJZ—Ganjingzi district; JZ—Jinzhou district; LSK—Lyushunkou district; PLD—Pulandian district; WFD—Wafangdian district; ZH—Zhuandhe district.
